# Supplementary material for: A Likelihood Approach for Real-Time Calibration of Stochastic Compartmental Epidemic Models
Source: PLoS Comput Biol. 2017 Jan 17;13(1):e1005257. doi: 10.1371/journal.pcbi.1005257 (PMC5240920; doi:10.1371/journal.pcbi.1005257)
Supplement: S1 File — (TAR.GZ) [file pcbi.1005257.s014.tar.gz › HSPH_Online-SI-Revision/Additions/IPoi10/n100-extreme-SEITR_noisy/n100-extreme-SEITR_noisy_BENCH2_table0.pdf]

1	newly 3 weeks spec	6225.94	6688.65	{4270., 9710.}	25.6499	0.106427	newly 3 weeks cumul	27 440.5	22 918.9	{16 635., 28 880.}	18.6592	0.0965724	newly 5 weeks spec	2726.36	4545.87	{2588., 7442.}	70.4147	0.205104	newly 5 weeks cumul	34 535.3	33 065.5	{22 642., 45 057.}	20.1529	0.0938934	newly 50 weeks cumul	41 541.4	48 853.5	{30 781., 73 187.}	31.0261	0.116437
2	newly 3 weeks spec	7321.	9317.59	{5398., 14 725.}	38.4758	0.135062	newly 3 weeks cumul	36 564.	35 450.2	{24 103., 45 708.}	17.8842	0.0840485	newly 5 weeks spec	2664.	4695.34	{2298., 7844.}	81.6125	0.210164	newly 5 weeks cumul	43 633.	46 905.5	{30 957., 64 828.}	22.3955	0.0926404	newly 58 weeks cumul	47 260.	55 499.6	{34 536., 79 283.}	29.6697	0.111819
3	newly 3 weeks spec	8747.	4285.9	{2368., 6788.}	51.9884	0.34756	newly 3 weeks cumul	40 258.	23 439.6	{14 028., 35 629.}	42.6054	0.263274	newly 5 weeks spec	3413.	1366.24	{735., 2252.}	60.4815	0.437229	newly 5 weeks cumul	49 075.	27 249.4	{16 112., 41 834.}	45.2086	0.285016	newly 60 weeks cumul	54 208.	28 930.3	{16 975., 44 593.}	47.3089	0.302748
4	newly 3 weeks spec	8496.	4879.49	{2443., 8099.}	48.6709	0.309397	newly 3 weeks cumul	36 998.	22 860.2	{12 691., 35 356.}	41.0488	0.249704	newly 5 weeks spec	3304.	1918.	{890., 3220.}	51.5066	0.327696	newly 5 weeks cumul	45 863.	27 868.1	{14 984., 43 365.}	43.09	0.264709	newly 58 weeks cumul	50 745.	30 861.9	{16 314., 49 059.}	43.9039	0.26997
5	newly 3 weeks spec	5120.	8185.99	{4422., 13 996.}	66.6947	0.190718	newly 3 weeks cumul	31 803.	36 664.7	{24 029., 53 225.}	28.5598	0.110233	newly 5 weeks spec	1318.	3057.58	{1365., 5974.}	134.014	0.286031	newly 5 weeks cumul	35 687.	44 841.6	{27 937., 66 613.}	36.1266	0.128293	newly 60 weeks cumul	37 045.	48 831.	{29 651., 79 558.}	41.1725	0.139448
6	newly 3 weeks spec	4778.	7692.83	{4113., 13 048.}	67.7756	0.194902	newly 3 weeks cumul	31 868.	33 689.7	{22 205., 47 426.}	23.6601	0.100558	newly 5 weeks spec	1057.	3018.38	{1361., 5021.}	185.837	0.379921	newly 5 weeks cumul	35 197.	41 667.9	{25 971., 63 461.}	32.2017	0.121302	newly 59 weeks cumul	35 989.	45 901.7	{27 898., 70 761.}	38.7981	0.135976
7	newly 3 weeks spec	7357.	5877.39	{3487., 8638.}	30.3383	0.164826	newly 3 weeks cumul	28 195.	20 792.9	{13 491., 29 112.}	27.9986	0.158541	newly 5 weeks spec	3977.	3899.63	{2006., 6154.}	40.7278	0.182951	newly 5 weeks cumul	37 670.	29 515.	{18 343., 41 828.}	28.1203	0.154502	newly 51 weeks cumul	47 209.	42 666.8	{24 044., 63 406.}	32.8621	0.160311
8	newly 3 weeks spec	7216.	7686.66	{4673., 13 200.}	32.7169	0.132278	newly 3 weeks cumul	32 722.	33 348.	{23 451., 47 725.}	21.5286	0.0928197	newly 5 weeks spec	3068.	3099.74	{1773., 5563.}	37.753	0.160044	newly 5 weeks cumul	40 537.	41 434.2	{28 250., 62 254.}	24.3449	0.103906	newly 58 weeks cumul	46 063.	46 151.3	{31 023., 70 975.}	25.634	0.111196
9	newly 3 weeks spec	5164.	7654.66	{4182., 12 207.}	55.3385	0.166503	newly 3 weeks cumul	32 212.	35 909.7	{23 204., 50 359.}	25.7944	0.102591	newly 5 weeks spec	1330.	2688.36	{1335., 4261.}	103.078	0.247856	newly 5 weeks cumul	36 196.	43 221.7	{26 945., 63 997.}	31.3909	0.116087	newly 60 weeks cumul	37 584.	46 609.1	{28 490., 69 288.}	34.6078	0.123046
10	newly 3 weeks spec	8694.	5267.18	{2869., 8678.}	43.9897	0.275076	newly 3 weeks cumul	33 638.	21 683.1	{13 292., 33 780.}	37.3686	0.22438	newly 5 weeks spec	4707.	2602.07	{1303., 4412.}	50.6213	0.332497	newly 5 weeks cumul	44 803.	28 016.1	{16 555., 44 688.}	40.2271	0.245634	newly 56 weeks cumul	56 292.	33 475.7	{19 387., 54 242.}	43.568	0.272192
11	newly 3 weeks spec	8565.	7129.61	{4046., 11 859.}	32.3512	0.168453	newly 3 weeks cumul	34 772.	26 504.9	{17 280., 37 176.}	25.9981	0.143517	newly 5 weeks spec	4255.	4026.2	{1941., 7263.}	39.7354	0.183096	newly 5 weeks cumul	45 069.	35 988.1	{22 105., 54 512.}	28.0805	0.151061	newly 55 weeks cumul	54 734.	45 737.	{26 677., 72 638.}	30.6661	0.158983
12	newly 3 weeks spec	7136.	7133.28	{4050., 12 298.}	31.8128	0.13969	newly 3 weeks cumul	31 568.	28 250.8	{18 464., 40 593.}	22.626	0.112203	newly 5 weeks spec	3139.	3543.18	{1828., 6562.}	40.7762	0.155042	newly 5 weeks cumul	39 318.	36 903.5	{23 032., 57 411.}	25.5201	0.120412	newly 56 weeks cumul	44 760.	44 093.1	{26 634., 71 358.}	27.9162	0.124821
13	newly 3 weeks spec	7637.	6284.87	{3513., 10 773.}	34.1655	0.178613	newly 3 weeks cumul	33 066.	26 583.3	{16 931., 41 198.}	27.6472	0.148244	newly 5 weeks spec	3594.	2861.12	{1522., 5196.}	39.6085	0.210063	newly 5 weeks cumul	41 882.	33 744.4	{20 862., 54 161.}	29.8802	0.159353	newly 57 weeks cumul	49 021.	39 028.7	{23 632., 64 051.}	31.7201	0.170011
14	newly 3 weeks spec	8364.	5455.	{2935., 8478.}	36.234	0.222752	newly 3 weeks cumul	30 226.	19 329.1	{11 256., 27 527.}	36.0515	0.215669	newly 5 weeks spec	4850.	3609.04	{1757., 5956.}	37.4285	0.217237	newly 5 weeks cumul	41 750.	27 404.7	{15 222., 40 548.}	34.8334	0.210908	newly 50 weeks cumul	54 343.	39 832.3	{20 641., 62 480.}	33.6891	0.196248
15	newly 3 weeks spec	7352.	5876.61	{3767., 9570.}	30.7676	0.163754	newly 3 weeks cumul	31 019.	20 883.4	{14 717., 30 249.}	32.6893	0.187293	newly 5 weeks spec	3380.	3868.74	{2121., 6870.}	42.0672	0.157135	newly 5 weeks cumul	39 368.	29 573.6	{19 633., 45 756.}	29.6127	0.163495	newly 51 weeks cumul	45 493.	42 334.4	{26 190., 69 435.}	31.4851	0.14674
16	newly 3 weeks spec	8547.	4522.4	{2461., 6975.}	49.7666	0.327761	newly 3 weeks cumul	32 656.	18 159.5	{10 683., 26 821.}	45.1598	0.287801	newly 5 weeks spec	4015.	2397.2	{1188., 3640.}	47.5751	0.30025	newly 5 weeks cumul	42 788.	23 884.5	{13 609., 35 464.}	46.0499	0.294523	newly 54 weeks cumul	49 793.	29 697.3	{16 448., 44 233.}	44.103	0.276012
17	newly 3 weeks spec	6343.	8199.53	{4145., 13 656.}	47.1199	0.15572	newly 3 weeks cumul	39 322.	36 857.7	{23 140., 52 621.}	23.4336	0.110889	newly 5 weeks spec	1376.	2929.04	{1329., 4994.}	116.116	0.266067	newly 5 weeks cumul	43 673.	44 807.4	{26 808., 68 231.}	27.981	0.120209	newly 60 weeks cumul	44 678.	48 526.8	{28 447., 74 667.}	30.5349	0.123028
18	newly 3 weeks spec	6778.	7220.83	{4146., 11 348.}	33.8407	0.134315	newly 3 weeks cumul	35 236.	33 042.5	{22 522., 46 980.}	21.9097	0.102224	newly 5 weeks spec	2583.	2677.05	{1439., 4221.}	39.6313	0.157981	newly 5 weeks cumul	42 014.	40 175.3	{26 508., 58 505.}	24.543	0.111317	newly 59 weeks cumul	45 747.	43 879.	{28 380., 64 408.}	25.8459	0.116227
19	newly 3 weeks spec	8875.	7717.61	{5361., 11 520.}	23.2531	0.114441	newly 3 weeks cumul	35 227.	27 759.8	{21 009., 35 349.}	21.6571	0.114857	newly 5 weeks spec	4524.	4711.96	{2787., 7650.}	33.0326	0.133134	newly 5 weeks cumul	46 101.	38 570.	{28 853., 52 581.}	20.7681	0.105033	newly 54 weeks cumul	56 361.	51 201.1	{35 759., 73 645.}	23.4039	0.110576
20	newly 3 weeks spec	6233.	7121.4	{4193., 10 276.}	35.4256	0.132654	newly 3 weeks cumul	33 857.	32 713.4	{22 850., 44 834.}	22.7309	0.110577	newly 5 weeks spec	2013.	2879.57	{1398., 4118.}	58.015	0.16168	newly 5 weeks cumul	39 540.	40 108.9	{26 741., 54 934.}	24.5827	0.105498	newly 59 weeks cumul	42 220.	44 747.2	{28 736., 63 974.}	27.5394	0.112129
21	newly 3 weeks spec	6457.	7860.37	{4404., 13 659.}	40.6649	0.146624	newly 3 weeks cumul	36 224.	36 171.	{24 182., 50 306.}	24.0457	0.109178	newly 5 weeks spec	1848.	3127.55	{1385., 4991.}	82.0933	0.19392	newly 5 weeks cumul	41 535.	44 312.9	{28 694., 66 142.}	26.6763	0.111132	newly 60 weeks cumul	43 675.	48 619.3	{30 347., 74 639.}	30.6022	0.120996
22	newly 3 weeks spec	7395.	3557.68	{1678., 6264.}	56.2626	0.39557	newly 3 weeks cumul	32 127.	15 529.	{7858., 27 227.}	52.9001	0.36795	newly 5 weeks spec	2882.	1658.01	{764., 3019.}	54.4822	0.352919	newly 5 weeks cumul	39 687.	19 632.5	{9656., 34 707.}	53.2634	0.368111	newly 56 weeks cumul	44 378.	22 938.8	{11 077., 40 644.}	52.8729	0.359012
23	newly 3 weeks spec	4881.	8186.77	{4993., 12 368.}	71.9856	0.223344	newly 3 weeks cumul	26 601.	32 498.2	{22 918., 43 207.}	30.5728	0.128928	newly 5 weeks spec	1370.	3981.49	{2137., 6269.}	190.62	0.42396	newly 5 weeks cumul	30 613.	42 257.9	{28 248., 59 063.}	43.7099	0.153934	newly 57 weeks cumul	31 913.	50 903.2	{33 089., 72 220.}	61.1484	0.191454
24	newly 3 weeks spec	7357.	10 554.9	{6596., 15 514.}	47.8972	0.155626	newly 3 weeks cumul	40 276.	40 485.3	{31 532., 48 237.}	12.9082	0.0573958	newly 5 weeks spec	2182.	4621.35	{2587., 7267.}	112.511	0.286468	newly 5 weeks cumul	46 540.	52 281.2	{38 106., 67 594.}	21.0903	0.0838638	newly 59 weeks cumul	48 960.	59 501.2	{42 007., 78 763.}	28.0532	0.103926
25	newly 3 weeks spec	9005.	8534.91	{5592., 13 119.}	24.2348	0.113053	newly 3 weeks cumul	36 976.	29 880.2	{21 369., 37 023.}	19.5293	0.102646	newly 5 weeks spec	3489.	5230.84	{2902., 9056.}	58.1932	0.174012	newly 5 weeks cumul	46 402.	41 943.5	{29 853., 56 626.}	18.9526	0.0927627	newly 55 weeks cumul	51 783.	55 266.9	{36 621., 81 238.}	24.8307	0.102338
26	newly 3 weeks spec	6466.	8248.14	{4589., 11 511.}	38.8673	0.142277	newly 3 weeks cumul	27 532.	27 850.	{18 686., 33 939.}	17.5946	0.0792892	newly 5 weeks spec	2648.	5363.88	{2369., 8702.}	105.903	0.279251	newly 5 weeks cumul	34 415.	40 013.8	{24 457., 51 682.}	27.8907	0.110416	newly 53 weeks cumul	38 357.	55 363.9	{30 790., 78 296.}	51.6836	0.172769
27	newly 3 weeks spec	7481.	4662.31	{2578., 7851.}	44.9701	0.277236	newly 3 weeks cumul	29 075.	17 991.2	{10 845., 28 804.}	40.6088	0.248905	newly 5 weeks spec	4125.	2660.69	{1383., 4594.}	48.0317	0.290351	newly 5 weeks cumul	38 835.	24 190.6	{14 087., 39 544.}	42.3064	0.259269	newly 53 weeks cumul	49 190.	31 269.8	{17 543., 51 657.}	43.7125	0.265771
28	newly 3 weeks spec	5303.	5371.61	{2604., 9063.}	39.7345	0.167371	newly 3 weeks cumul	34 205.	26 973.2	{15 426., 43 687.}	32.1009	0.173686	newly 5 weeks spec	965.	1799.82	{831., 3048.}	93.3333	0.2272	newly 5 weeks cumul	37 460.	31 940.7	{17 712., 52 224.}	32.5493	0.165266	newly 60 weeks cumul	38 276.	34 208.2	{18 780., 56 185.}	32.6971	0.159116
29	newly 3 weeks spec	9186.	3987.36	{2107., 6845.}	59.8914	0.427974	newly 3 weeks cumul	36 850.	17 822.9	{10 152., 29 780.}	52.8377	0.357366	newly 5 weeks spec	4278.	1762.34	{870., 2969.}	63.8166	0.468769	newly 5 weeks cumul	47 405.	22 269.5	{12 445., 37 316.}	55.0405	0.377692	newly 57 weeks cumul	55 913.	25 484.8	{13 922., 42 820.}	56.8368	0.395483
30	newly 3 weeks spec	9408.	10 733.8	{6749., 16 034.}	30.3781	0.117352	newly 3 weeks cumul	43 928.	42 859.1	{33 572., 51 989.}	13.9049	0.0643374	newly 5 weeks spec	3519.	4418.53	{2463., 7093.}	44.9844	0.150209	newly 5 weeks cumul	53 150.	54 360.6	{40 635., 70 112.}	17.3087	0.075233	newly 60 weeks cumul	58 223.	60 703.	{43 877., 80 837.}	19.9829	0.0846378
31	newly 3 weeks spec	6334.	6072.55	{3687., 9442.}	27.9427	0.129206	newly 3 weeks cumul	24 601.	21 159.7	{14 428., 28 976.}	22.0777	0.113694	newly 5 weeks spec	3288.	4082.96	{2162., 6987.}	44.8501	0.15937	newly 5 weeks cumul	32 650.	30 282.9	{19 460., 44 428.}	24.4129	0.116811	newly 50 weeks cumul	39 652.	44 585.5	{26 235., 69 279.}	34.3087	0.134813
32	newly 3 weeks spec	8242.	6681.64	{3581., 10 033.}	32.5074	0.174988	newly 3 weeks cumul	32 101.	26 323.	{16 193., 36 729.}	25.0845	0.133774	newly 5 weeks spec	4308.	3488.88	{1655., 5304.}	41.016	0.215211	newly 5 weeks cumul	42 469.	34 713.4	{20 187., 50 120.}	27.8651	0.149339	newly 56 weeks cumul	52 787.	42 246.5	{23 433., 61 257.}	31.0577	0.167668
33	newly 3 weeks spec	5633.	1784.78	{930., 2898.}	70.4304	0.572216	newly 3 weeks cumul	30 340.	10 910.6	{5696., 17 604.}	66.0315	0.5077	newly 5 weeks spec	2012.	507.734	{265., 837.}	75.678	0.669513	newly 5 weeks cumul	35 798.	12 371.1	{6439., 20 000.}	67.2897	0.525703	newly 60 weeks cumul	38 479.	12 942.1	{6713., 20 923.}	68.0627	0.537493
34	newly 3 weeks spec	6383.	6042.09	{3414., 11 015.}	35.7504	0.160769	newly 3 weeks cumul	29 792.	24 581.5	{15 761., 39 020.}	28.6583	0.147795	newly 5 weeks spec	2446.	2971.56	{1584., 5680.}	46.7596	0.158217	newly 5 weeks cumul	36 225.	31 843.1	{19 588., 53 693.}	30.5109	0.148652	newly 56 weeks cumul	39 694.	37 855.7	{22 586., 65 307.}	31.8676	0.14314
35	newly 3 weeks spec	5611.	7291.16	{4296., 12 012.}	44.4168	0.149249	newly 3 weeks cumul	36 155.	35 345.	{24 609., 51 561.}	24.6561	0.111126	newly 5 weeks spec	1151.	2447.28	{1317., 4254.}	113.092	0.281949	newly 5 weeks cumul	39 920.	42 073.1	{28 349., 63 130.}	27.8954	0.116222	newly 60 weeks cumul	40 865.	45 154.7	{30 018., 68 466.}	29.9039	0.118541
36	newly 3 weeks spec	9648.	9367.02	{5951., 13 376.}	24.6657	0.11346	newly 3 weeks cumul	37 219.	32 669.7	{25 023., 39 080.}	14.5233	0.0726946	newly 5 weeks spec	5029.	5402.	{2811., 8846.}	39.3066	0.161169	newly 5 weeks cumul	49 093.	45 372.	{32 159., 57 761.}	17.5265	0.0845016	newly 56 weeks cumul	60 543.	57 518.7	{38 180., 78 570.}	22.4627	0.104816
37	newly 3 weeks spec	8031.	9765.18	{6281., 14 685.}	32.0615	0.117897	newly 3 weeks cumul	39 903.	34 998.1	{27 411., 42 140.}	14.3515	0.0709893	newly 5 weeks spec	2840.	5281.71	{2969., 9199.}	86.9588	0.234942	newly 5 weeks cumul	47 625.	47 673.6	{34 720., 60 669.}	17.3414	0.0769826	newly 57 weeks cumul	51 821.	58 765.7	{40 808., 82 228.}	24.8988	0.0969679
38	newly 3 weeks spec	6443.	6142.91	{3554., 9318.}	30.0241	0.138833	newly 3 weeks cumul	29 746.	23 269.4	{15 188., 33 058.}	25.9221	0.139793	newly 5 weeks spec	2643.	3546.35	{1806., 5626.}	51.2176	0.162785	newly 5 weeks cumul	36 633.	31 528.3	{19 569., 45 891.}	26.2781	0.133292	newly 54 weeks cumul	40 381.	40 645.5	{23 955., 61 315.}	30.571	0.132482
39	newly 3 weeks spec	8056.	6304.71	{3626., 10 089.}	32.7227	0.174916	newly 3 weeks cumul	34 505.	25 332.6	{16 173., 36 894.}	29.358	0.162628	newly 5 weeks spec	3317.	3182.16	{1645., 5257.}	35.1582	0.15749	newly 5 weeks cumul	43 007.	33 063.	{20 326., 49 530.}	29.6534	0.160381	newly 56 weeks cumul	48 356.	39 858.8	{23 806., 61 160.}	28.8364	0.149217
40	newly 3 weeks spec	7422.	3818.94	{1929., 7373.}	54.489	0.370123	newly 3 weeks cumul	30 770.	17 143.8	{9135., 32 005.}	48.3765	0.314166	newly 5 weeks spec	3674.	1660.62	{773., 3337.}	60.1079	0.43446	newly 5 weeks cumul	39 721.	21 348.9	{11 109., 40 470.}	50.5998	0.334748	newly 57 weeks cumul	48 338.	24 402.9	{12 514., 46 538.}	53.2191	0.362529
41	newly 3 weeks spec	7711.	11 849.8	{7377., 18 181.}	56.8295	0.178597	newly 3 weeks cumul	45 986.	44 927.9	{35 916., 52 423.}	11.8999	0.0543469	newly 5 weeks spec	1635.	4887.89	{2543., 8450.}	198.954	0.430952	newly 5 weeks cumul	51 212.	57 709.	{42 968., 71 585.}	20.9851	0.0835717	newly 60 weeks cumul	52 636.	64 677.2	{46 592., 84 913.}	28.5327	0.105576
42	newly 3 weeks spec	7808.	5883.6	{3803., 7823.}	27.4811	0.151004	newly 3 weeks cumul	27 957.	21 337.3	{15 031., 27 393.}	23.9792	0.129338	newly 5 weeks spec	4650.	3728.12	{2253., 5007.}	30.8817	0.160677	newly 5 weeks cumul	38 862.	29 804.6	{20 260., 38 760.}	24.9809	0.135496	newly 51 weeks cumul	51 353.	41 762.1	{26 836., 54 823.}	26.0755	0.135988
43	newly 3 weeks spec	8961.	6051.07	{3197., 10 272.}	42.9126	0.254551	newly 3 weeks cumul	34 781.	23 411.4	{14 474., 36 645.}	34.9638	0.205014	newly 5 weeks spec	4598.	3271.9	{1501., 6562.}	52.6526	0.298852	newly 5 weeks cumul	46 123.	31 203.9	{18 104., 49 748.}	37.6612	0.221912	newly 56 weeks cumul	56 656.	38 452.7	{21 106., 68 907.}	41.6169	0.244252
44	newly 3 weeks spec	7188.	8855.72	{5870., 13 465.}	32.9058	0.119658	newly 3 weeks cumul	31 836.	32 574.3	{25 027., 40 394.}	15.7405	0.0686915	newly 5 weeks spec	3246.	4767.63	{2705., 8274.}	53.8872	0.165022	newly 5 weeks cumul	39 959.	43 984.8	{31 930., 59 601.}	21.4333	0.0867065	newly 56 weeks cumul	46 072.	54 379.8	{37 791., 78 338.}	27.7703	0.104433
45	newly 3 weeks spec	7528.	7132.45	{3921., 12 718.}	36.8789	0.170574	newly 3 weeks cumul	33 385.	31 144.9	{19 760., 47 550.}	26.1456	0.124377	newly 5 weeks spec	3187.	2861.59	{1438., 5496.}	43.1825	0.207935	newly 5 weeks cumul	41 374.	38 611.2	{23 548., 61 490.}	29.0837	0.137548	newly 58 weeks cumul	46 827.	42 953.6	{25 486., 69 707.}	30.6346	0.146754
46	newly 3 weeks spec	8312.	3332.11	{1488., 6576.}	65.6788	0.503885	newly 3 weeks cumul	39 284.	17 644.1	{8485., 34 264.}	58.0337	0.418849	newly 5 weeks spec	3124.	1078.81	{431., 2063.}	71.1903	0.584826	newly 5 weeks cumul	47 611.	20 624.2	{9685., 39 988.}	60.0919	0.441125	newly 60 weeks cumul	52 255.	21 961.	{10 213., 42 572.}	61.3371	0.456318
47	newly 3 weeks spec	8223.	6897.43	{4074., 11 547.}	32.5679	0.16678	newly 3 weeks cumul	34 349.	26 160.9	{17 727., 37 341.}	26.8044	0.146898	newly 5 weeks spec	3657.	3798.67	{2024., 7107.}	39.5315	0.165402	newly 5 weeks cumul	43 642.	35 156.6	{22 660., 54 198.}	28.4775	0.150633	newly 55 weeks cumul	49 521.	44 148.5	{27 534., 71 409.}	29.5661	0.144205
48	newly 3 weeks spec	8405.	7569.79	{4232., 11 118.}	27.5449	0.134874	newly 3 weeks cumul	39 417.	30 635.8	{19 688., 41 430.}	24.5172	0.136075	newly 5 weeks spec	2763.	3696.76	{1805., 5356.}	49.9074	0.160891	newly 5 weeks cumul	47 174.	39 661.4	{24 603., 54 678.}	23.6431	0.122577	newly 58 weeks cumul	50 734.	47 036.9	{27 869., 65 536.}	24.8916	0.118617
49	newly 3 weeks spec	6054.	3733.77	{1944., 6361.}	48.6062	0.295299	newly 3 weeks cumul	27 886.	16 414.1	{9178., 26 903.}	46.3906	0.28781	newly 5 weeks spec	2454.	1717.02	{880., 2852.}	47.9693	0.269793	newly 5 weeks cumul	34 341.	20 673.6	{11 358., 34 068.}	46.7421	0.287089	newly 56 weeks cumul	38 035.	24 062.	{13 004., 39 838.}	46.0787	0.275807
50	newly 3 weeks spec	8264.	6088.23	{3197., 9844.}	39.276	0.221559	newly 3 weeks cumul	33 965.	25 609.	{15 248., 39 002.}	31.1112	0.174091	newly 5 weeks spec	3752.	2804.1	{1321., 4566.}	44.7889	0.248141	newly 5 weeks cumul	43 375.	32 599.4	{18 566., 50 377.}	33.7733	0.188775	newly 57 weeks cumul	50 179.	37 788.2	{20 939., 58 672.}	35.4814	0.197481
